# Assessing Functional Outcomes and Complications After Turndown Flap With Flexor Hallucis Longus Augmentation in Chronic Achilles Tendon Ruptures

**DOI:** 10.7759/cureus.106610

**Published:** 2026-04-07

**Authors:** Sharina Mohd Khalid, Mohd Fadhli Miskon

**Affiliations:** 1 Orthopaedic Surgery, Hospital Sultan Ismail, Johor Bahru, MYS; 2 Orthopaedics and Traumatology, Hospital Sultan Ismail, Johor Bahru, MYS

**Keywords:** chronic achilles tendon rupture, complications, flexor hallucis longus (fhl) transfer, functional outcome, turndown flap

## Abstract

Introduction: Chronic Achilles tendon rupture (CATR) presents a therapeutic challenge due to tendon retraction, degeneration, and muscle atrophy that typically occur beyond four weeks after injury, complicating delayed repair. To address the resultant tendon defect and restore functional continuity, several reconstructive strategies have been described, including turndown flap augmentation and flexor hallucis longus (FHL) tendon transfer.

Objective: This study evaluated functional outcomes and complications following combined turndown flap augmentation and FHL tendon transfer for CATR.

Methodology: This retrospective study included eight patients with CATR who underwent turndown flap augmentation combined with FHL tendon transfer between March 2018 and December 2024. Chronic rupture was confirmed clinically and radiographically. Functional outcomes were assessed using the Foot and Ankle Ability Measure (FAAM) and the Achilles Tendon Rupture Score (ATRS) preoperatively and at follow-up. Complications, operative time, and postoperative outcomes were recorded and analyzed.

Results: Eight patients (mean age, 53 ± 13.3 years; males, 5 (62.5%); females, 3 (37.5%)) were included. Four of the patients (50%) had a sedentary lifestyle, while another four (50%) were active in sports. Three patients (37.5%) had diabetes mellitus, while five (62.5%) had no comorbidities. A history of previous tendinitis was found in two patients (25.0%). Intraoperatively, tendinitis with calcification was found in four patients (50%). The mean tendon gap was 5.25 ± 1.58 cm, and the mean length of the distal stump from the tendo-Achilles insertion was 1.75 cm. The mean operative time was 122 ± 20 minutes. Reconstruction was performed at a median of 4 months after injury, with a mean follow-up of 13 months (range, 6-36 months). Significant functional improvement was observed. FAAM Activities of Daily Living scores improved from 49.6% ± 14.9% preoperatively to 83.5% ± 16.6% postoperatively (*P* = 0.0005), and FAAM Sports scores improved from 22.3% ± 13.1% to 71.5% ± 29.9% (*P* = 0.0002). ATRS improved from 31.4% ± 16.9% to 82.5% ± 12.8% (*P* < 0.001). All patients were able to perform a single-limb heel rise. Complications were limited, with one case each of wound dehiscence (12.5%), transient neuropraxia (12.5%), and complex regional pain syndrome (12.5%). No neurovascular injury or tendon re-rupture occurred.

Conclusions: Turndown flap augmentation combined with FHL tendon transfer provides significant functional improvement with acceptable complications in the treatment of CATR. This technique represents a reliable option for the reconstruction of large tendon defects in delayed presentations.

## Introduction

Chronic Achilles tendon rupture (CATR) occurs in approximately 20%-25% of patients, most often as a result of missed diagnosis or delayed treatment beyond four weeks after injury [1]. Patients commonly present with gait impairment, reduced push-off strength, and functional limitation, emphasizing the need for effective reconstruction.

Achilles tendon ruptures most commonly occur 1-6 cm proximal to the tendon’s insertion at the calcaneal tuberosity. It poses a significant challenge in surgical treatment due to the lack of viable tendinous tissue of the paratenon at the rupture site. Furthermore, the interval between the proximal and distal tendon stumps is typically occupied by dense scar tissue [2,3]. Management of these complex injuries has evolved substantially over recent decades, progressing from primary end-to-end repair to advanced reconstructive techniques incorporating tendon transfers and augmentation flaps [4].

Direct end-to-end repair is suitable for acute ruptures but is often not feasible in chronic cases because of large tendon defects, typically exceeding 3-6 cm [5]. In these settings, primary repair is often performed under excessive tension, increasing the risk of re-rupture and compromising functional outcomes. In addition, degenerative tendon changes reduce intrinsic healing capacity, limiting the durability of conventional repair. These limitations underscore the need for augmentation or tendon transfer techniques to achieve reliable reconstruction in chronic rupture. Multiple reconstructive techniques have been described for managing tendon defects, including V-Y advancement, turndown flap augmentation, and tendon transfer procedures [5,6]. Nevertheless, the lack of prospective randomized trials and small study cohorts precludes a standardized approach.

Among these techniques, flexor hallucis longus (FHL) tendon transfer is commonly preferred because of its anatomical proximity, similar line of pull, and ability to restore plantarflexion strength [7]. FHL tendon transfer also offers the advantage of a highly vascularised muscle belly that may enhance tendon healing [8]. Gastrocnemius turndown fascial flaps provide a strong local graft aligned with the native tendon, facilitating restoration of normal ankle biomechanics [9]. Turndown flap augmentation provides additional autologous tissue, enhancing both biological healing and mechanical integrity. In combination, these techniques offer complementary benefits in which FHL provides vascularity while the turndown flap provides structural length that may improve repair durability and enhance functional outcomes.

The purpose of this case series was to describe the management and short-term outcomes of eight patients with CATR treated with combined turndown flap augmentation and FHL tendon transfer, thereby contributing additional cohort data to the limited existing literature.

## Materials and methods

This single-center retrospective study was conducted at Hospital Sultan Ismail, Johor Bahru, Malaysia. Patients who underwent gastrocnemius turndown flap augmentation combined with FHL tendon transfer between March 2018 and December 2024 were identified. Eight patients who met the inclusion criteria were included in the series. Inclusion criteria comprised adult patients with CATR who underwent gastrocnemius turndown flap augmented with FHL tendon transfer. Exclusion criteria included (1) open Achilles tendon ruptures, (2) concomitant ruptures with fractures, (3) tendon gap defects less than 2 cm, (4) injuries of less than four weeks’ duration, and (5) compromised skin condition at the surgical site.

**Figure 1:**
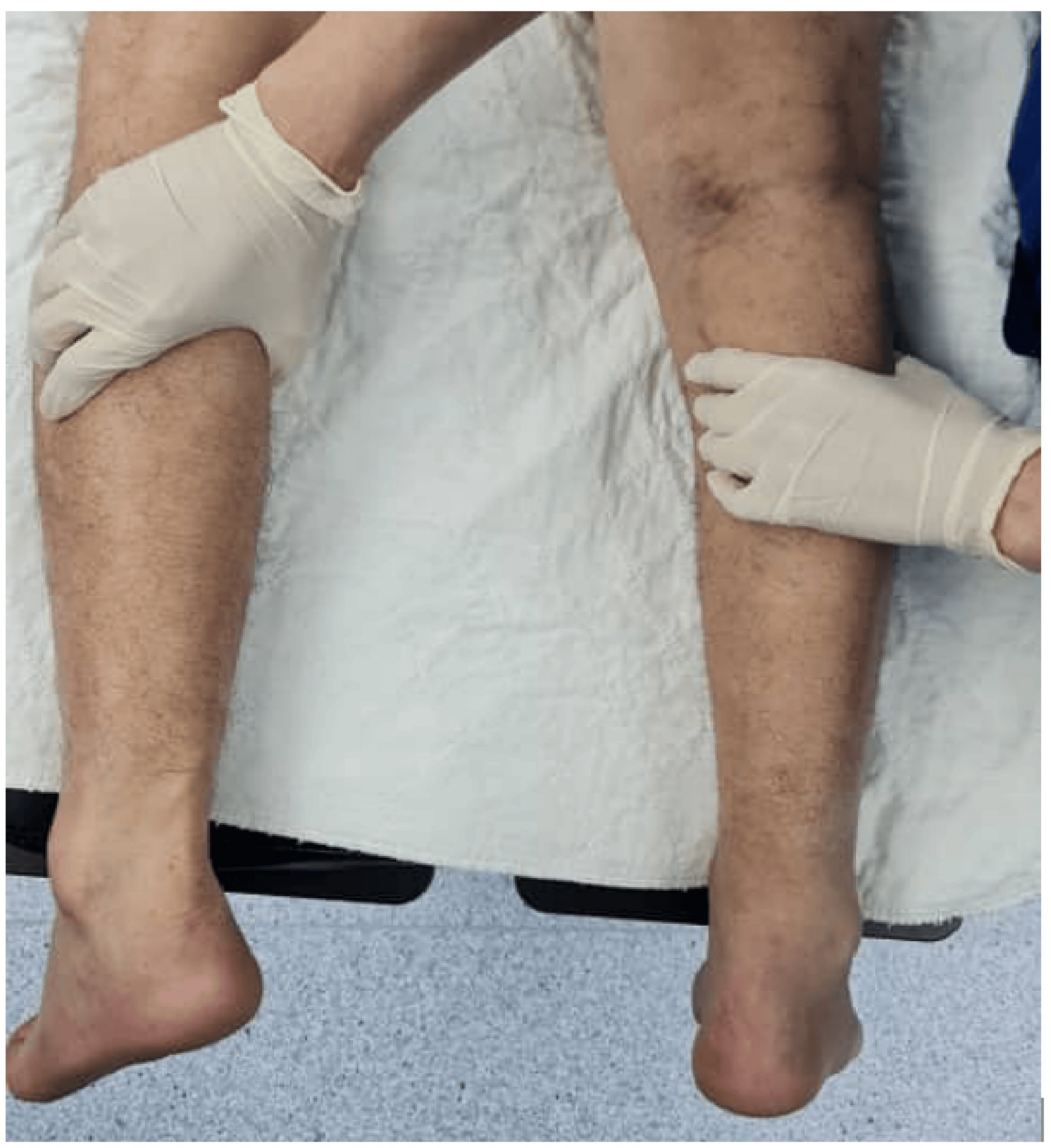
Simmonds-Thompson test. With the patient in the prone position and the foot hanging freely, calf compression produces little or no plantarflexion in cases of Achilles tendon rupture.

All procedures were performed by a single surgeon, and a standardized postoperative rehabilitation protocol was applied. Diagnosis was established through clinical examination, including palpation for a gap and the Simmonds-Thompson test (Figure 1). Patients were also unable to perform a single-leg heel rise. The diagnosis was further confirmed with radiological imaging, either by ultrasound or magnetic resonance imaging. Functional outcomes were assessed preoperatively and at follow-up using the Foot and Ankle Ability Measure (FAAM) [10] and the Achilles Tendon Rupture Score (ATRS) [11]. Operative duration and postoperative complications, including wound-related problems, donor-site morbidity, and tendon re-rupture, were recorded and analyzed.

Statistical analysis

All statistical analyses were performed using VassarStats online statistical computation tools (Lowry 2001-2024). Paired t-tests were conducted to compare preoperative and postoperative FAAM and ATRS scores. Statistical significance was set at *P *< 0.05.

Surgical technique

Preoperatively, all patients underwent routine laboratory investigations and cardiopulmonary and anesthetic assessments to confirm medical fitness for surgery. Procedure-related risks were explained, and informed consent was obtained. Surgery was performed under combined spinal-epidural anesthesia (CSE), with prophylactic antibiotics administered at induction. Patients were positioned prone with the ipsilateral foot free and unsupported at the end of the operating table. A thigh tourniquet was applied and inflated to 300 mmHg. The operative field was then prepared and draped in a sterile manner.

**Figure 2:**
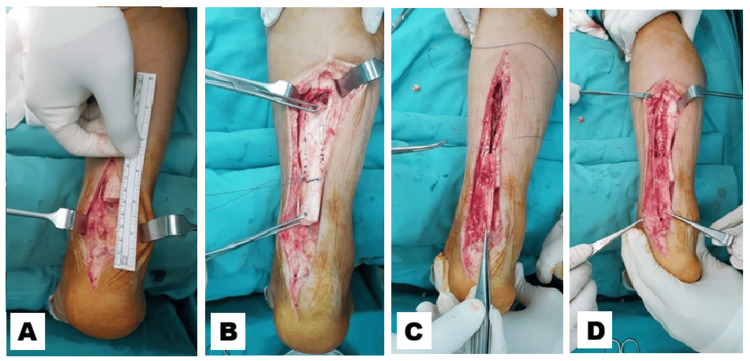
Degenerated tendon tissue was excised and the defect measured (A), followed by creation of a gastrocnemius turndown flap based on the defect length (B).

A longitudinal skin incision was made over the posterior aspect of the Achilles tendon. Dissection was carried through the subcutaneous tissue, fascia, and tendon sheath to expose the rupture site. Degenerated and scarred tendon tissue between the ruptured ends was excised to healthy margins (Figure 2A), after which the tendon defect was measured (Figure 2B). The gastrocnemius fascia was then marked according to the measured defect length and incised to create a turndown flap (Figure 2C).

**Figure 3:**
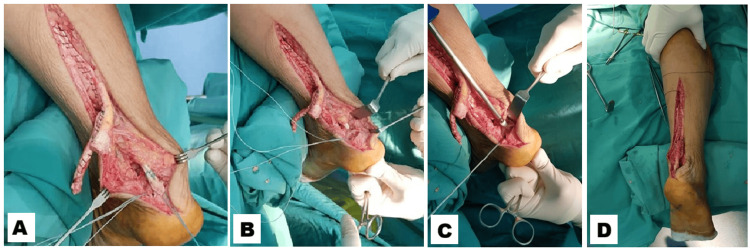
The flexor hallucis longus tendon was identified and harvested proximal to the knot of Henry (A) and anchored into the calcaneum with an interference screw (B-C). The fascial flap was then secured to the distal stump and reinforced with suture anchor fixation (D).

The fascial flap was harvested from the proximal tendon stump while maintaining distal continuity and rotated distally. The FHL tendon was subsequently identified and harvested proximal to the knot of Henry, then anchored into the calcaneum using an interference screw. The fascial flap harvested was secured to the distal stump at the calcaneal insertion using either direct end-to-end suturing and augmented with suture anchor fixation into the calcaneum. High-strength non-absorbable sutures such as No. 2 FiberWire and No. 5 Ethibond are commonly used for direct end-to-end suturing. Appropriate Achilles tendon tension was restored based on intraoperative clinical assessment and comparison with the contralateral side. Following copious irrigation with normal saline, layered wound closure was performed. A posterior plaster slab was applied with the ankle maintained in plantarflexion (Figure 3).

Postoperative follow-up

Postoperatively, the ankle was immobilized in a plantarflexed dorsal slab for two weeks. At two weeks, wound inspection and suture removal were performed, and immobilization was converted to a backslab at 30° of plantarflexion with continued non-weight-bearing and lower-limb strengthening exercises. At four to six weeks, patients were transitioned to an Aircast boot with heel raises, permitted partial weight-bearing, and commenced ankle range-of-motion exercises to neutral, resisted ankle strengthening, and proprioceptive training. At six weeks, partial weight-bearing in neutral and progression of dorsiflexion exercises were allowed. By eight weeks, full weight-bearing was permitted, with gradual progression of active ankle motion and continued strengthening and balance exercises.

## Results

Eight patients with CATR and tendon gaps greater than 4 cm underwent open reconstruction using a gastrocnemius-soleus turndown flap augmented with FHL tendon transfer. Five patients were male (62.5%), and three patients were female (37.5%). The mean age of the studied patients was 53 ± 13.3 years. All eight patients sustained a traumatic injury at presentation. Falls during routine daily activities were the most common mechanism, occurring in 4 (50%) patients. Three patients (37.5%) were injured during recreational sports, including badminton (2, 25%) and sepak takraw (1, 12.5%). One patient (12.5%) sustained the injury following a motor vehicle accident involving a fall from a motorcycle. Four patients had a sedentary lifestyle (50%), while another four (50%) were active in sports. Three patients (37.5%) had diabetes mellitus, while five (62.5%) had no comorbidities. Surgical delay was due to delayed presentation to medical care in five (62.5%) patients, initial misdiagnosis in one (12.5%), and failure of nonoperative management in two (25%), in which patients initially elected conservative treatment for Achilles tendon rupture. A history of previous tendinitis was found in two (25%) patients. Intraoperatively, tendinitis with calcification was observed in four (50%) patients (Table 1).

**Table 1:** Demographic and clinical characteristics of the patients (data presented as n (%) or mean ± SD; N = 8). SD, standard deviation

Variables	*n* (%)/Mean ± SD (*N* = 8)
Age (Years)	
Range	33-70 years
Mean	53.12 ± 13.13 years
Gender	
Male	5 (62.5%)
Female	3 (37.5%)
Side	
Right	4 (50%)
Left	4 (50%)
Cause of rupture (Trauma)	
Fall	4 (50%)
Sports related	3 (37.5%)
Motor vehicle accident	1 (12.5%)
Comorbidities	
None	5 (62.5%)
Diabetes mellitus	3 (37.5%)
Smoking status	
Non-smoker	7 (87.5%)
Smoker	1 (12.5%)
Insertional calcific Achilles tendinopathy (ICAT)	
Present	4 (50%)
None	4 (50%)
Percentage per total	

Reconstruction was performed at a median of four months (interquartile range, 2-6 months) following injury. The mean tendon gap measured intraoperatively was 5.25 cm, and the mean length of the distal stump from tendo-Achilles (TA) insertion was 1.75 cm. A history of previous tendinitis was found in two patients. Intraoperatively, tendinitis with calcification was observed in four patients (50%). The mean operative duration was 122 ± 20 minutes. The mean follow-up duration was 13 months (range, 6-36 months) (Table 2). There were no intraoperative complications.

**Table 2:** Operative data of the studied patients (data presented as n (%), mean ± SD; N = 8). SD, standard deviation

Variables	*n* (%)/Mean ± SD (*N *= 8)
Time to surgery	3.75 ± 1.39 months (range 2-6 months)
Length of gap	5.25 (±1.58) cm (range 4-9 cm)
Length of distal stump from TA insertion	1.75 (±1.04) cm
No stump	1 (12.5%)
1 cm	2 (25%)
2 cm	3 (37.5%)
3 cm	2 (25%)
Operative time (minutes)	122 ± 20 minutes (range 90-150 minutes)
Mean follow-up duration	13 months (range 6-36 months).

Functional outcomes improved significantly at final follow-up (Table 3). The mean FAAM Activities of Daily Living (ADL) score improved from 49.6% ± 14.9% preoperatively to 83.5% ± 16.6% postoperatively, representing a mean increase of 33.9% ± 15.7%. This improvement was statistically significant on paired analysis (*t* = 6.12, *P* = 0.0005). Similarly, the FAAM-Sports score improved from 22.3% preoperatively to 71.5% postoperatively, with a mean increase of 49.2% ± 19.2%, which was also statistically significant (*t* = 7.26, *P* = 0.0002). All patients achieved improvements exceeding the minimal clinically important difference for both subscales. Similarly, the ATRS demonstrated a marked improvement, increasing from 31.4% ± 16.9% preoperatively to 82.5% ± 12.8% postoperatively (*t* > 5.41, *P* < 0.001). All patients were able to perform a single-limb heel rise postoperatively.

**Table 3:** Functional scores of the patients (data presented as mean ± SD). ᵃ*P*-values were calculated using a paired t-test. Data are presented as mean ± SD. Statistical significance set at *P* < 0.05. SD, standard deviation

Variables	Preoperative (mean ± SD)	Postoperative (mean ± SD)	t-valueᵃ	*P*-valueᵃ
FAAM				
ADL Subscale	49.6% ± 14.9%	83.5% ± 16.6%	6.12ᵃ	0.0005ᵃ
Sports Subscale	22.3% ± 13.1%	71.5% ± 29.9%	7.26ᵃ	0.0002ᵃ
ATRS	31.4 ± 16.9	82.5 ± 12.8	5.41ᵃ	<0.001ᵃ

Complications were limited to three cases (37.5%). One patient developed wound dehiscence (12.5%), which resolved following surgical debridement, antibiotic therapy, and local wound care. This represented one of three patients within the diabetes subgroup. One patient developed dorsomedial foot pain postoperatively, likely related to transient neuropraxia of the saphenous nerve secondary to tourniquet use, which resolved by eight months. One patient developed complex regional pain syndrome, which improved with gabapentin treatment. No major neurovascular complications or Achilles tendon re-ruptures were observed.

## Discussion

CATR is not uncommon and is frequently missed, with up to 20% reported as clinically neglected [7]. CATR can result in significant gait impairment due to altered gastrocnemius-soleus mechanics and progressive muscle atrophy over time [12]. As a result, surgical management is generally recommended. However, treatment remains challenging, with no established standard approach.

Although surgical reconstruction can restore tendon strength and improve activity levels [5,12], chronic ruptures are technically demanding and may affect the choice of technique [13,14]. Debridement often enlarges the defect, making tendon transfer necessary. FHL transfer is commonly used due to its anatomical proximity, similar line of pull, and additional vascular support to the repair site [15,16,17]. Although FHL transfer may reduce hallux interphalangeal joint plantarflexion strength [18] and push-off [19], evidence suggests that overall lower-limb function is largely preserved, with no significant strength differences compared with the contralateral limb [19,20]. Given satisfactory functional recovery even in markedly delayed presentations, FHL transfer remains an appropriate option for CATR.

The gastrocnemius tendon is readily accessible and shares a similar line of pull with the Achilles tendon, allowing restoration of normal ankle biomechanics [21]. Ibrahim et al. [22] reported that the modified gastrocnemius turndown flap is a simple, safe, and effective technique with excellent functional outcomes and high patient satisfaction. In their series of 18 patients, complications were limited to two cases of superficial wound infection and two cases of mild ankle stiffness. Despite comparable functional outcomes across techniques, the choice of open surgical method influences complication risk in CATR, with turndown flaps associated with higher wound complication rates [4,17].

In our study, gastrocnemius fascial turndown flap with FHL augmentation produced favorable outcomes, with significant improvement in function, pain reduction, and return to daily activities. This combined approach is particularly useful for CATRs with large defects, especially when the tendon stumps are short or absent. When stump integrity is inadequate, FHL transfer can be performed by harvesting the tendon and passing it through bone tunnels in the posterior calcaneus to restore plantarflexion strength [13]. Nevertheless, preservation of the native Achilles stump remains important, as it maintains the normal tendon-bone interface and Sharpey’s fibers [23], whereas FHL transfer requires a longer period for interface remodeling and structural integration [24]. However, the relatively long mean operative time (122 minutes) may represent a potential drawback of this technique.

Tay et al. [25] reported satisfactory outcomes at two-year follow-up in patients with CATR treated using double turndown flaps with FHL augmentation. The mean post-debridement tendon gap in their series of nine patients was 5.6 cm (range 4-7 cm), comparable to our intraoperative mean gap of 5.3cm. They observed one case of sural neuropraxia and two cases of heel numbness, all of which resolved subsequently. In a similar study [26], 13 patients were treated with a gastrocnemius fascial turndown flap combined with FHL augmentation, with only one wound-related complication reported, which resolved with antibiotics and regular dressings. Koh et al [6] reported comparable outcomes with the use of a turndown flap combined with FHL augmentation. They further demonstrated that, in patients with similar demographics and clinical presentation, FHL transfer alone and turndown flap augmented with FHL transfer produced similar results at 12 months postoperatively.

The complication rate in our series (3, 37.5%) appears higher than that reported in the literature (approximately 14.4%) [17]. However, these findings should be interpreted with caution, given the small sample size, where a small number of events can disproportionately affect the overall percentage. A systematic review [17] reported that the optimal surgical technique for CATR remains controversial. Although surgery improves patient-reported outcomes at midterm follow-up, it carries a complication rate of 11.4%, most commonly superficial wound infection and sural nerve hypoesthesia. The same review found that gastrocnemius turndown fascial flaps provided good strength, preserved heel-raise function, and high return-to-play rates, supporting their use when end-to-end repair was not feasible [17]. For ruptures exceeding three months, FHL transfer is preferred for substitution and augmentation of the atrophied tendon, with compensatory hypertrophy and vascularity enabling strength comparable to the contralateral side [20]. Biologic reconstruction may reduce complications associated with synthetic grafts, which have shown a 20.0% complication rate, mainly due to superficial wound infection [17,22].

Our study is limited by its small sample size and retrospective design. There was no comparison with other repair techniques, and objective assessments such as gait analysis and isokinetic strength testing were not performed. Larger, multicenter randomized controlled trials with longer follow-up and comprehensive functional evaluation are needed to provide stronger evidence.

## Conclusions

Gastrocnemius turndown flap with FHL augmentation provides a reliable option for the treatment of CATRs. This study is exploratory in nature and aims to report the clinical outcomes of this combined technique in a small patient cohort. The procedure was associated with good clinical outcomes, improved functional performance, and high patient satisfaction, with an acceptable rate of complications. Further studies with larger cohorts and longer follow-up are required to better establish these findings.
